# Physiological and Behavioral Indicators to Measure Crustacean Welfare

**DOI:** 10.3390/ani9110914

**Published:** 2019-11-03

**Authors:** Rebecca Adams, Catherine E. Stanley, Elena Piana, Robin L. Cooper

**Affiliations:** 1 Department of Biology, University of Kentucky, Lexington, KY 40506, USA; adamsr11@mymail.nku.edu (R.A.); cest242@g.uky.edu (C.E.S.); 2 Department of Biological Sciences, Northern Kentucky University, Highland Heights, KY 41099, USA; 3 Sea Farms Limited, Redditch, Worcestershire B98 0RE, UK; EPiana@sea-farms.com

**Keywords:** crayfish, slaughter, heating

## Abstract

**Simple Summary:**

The purpose of this project was to determine how neural circuits were affected during warming by examining sensory neurons, the neuromuscular junction, and the cardiac function and behavior of the commercially important crustacean species, the red swamp crayfish (*Procambarus clarkii*). Various rates of heating at 1 °C/min, 12 °C/min, or 46 °C/min to 80 °C as well as placing crayfish directly in boiling water were examined. Sensory nerves and the neuromuscular junction will stop working at 44 °C within two minutes. The heart ceases functioning fastest (within 10 s) when placing the crayfish directly in boiling water, which is the quickest method to kill them while minimizing exposure to noxious stimuli.

**Abstract:**

This project determined how neural circuits are affected during warming by examining sensory neurons, the neuromuscular junction, and the cardiac function and behavior of the commercially important crustacean species, the red swamp crayfish (*Procambarus clarkii*). Rapid inactivation of neural function in crustaceans prior to slaughter is important to limit exposure to noxious stimuli, thus improving animal welfare. This study demonstrated that as a crayfish is warmed at 1 °C/min, the heart beat stops at 44 °C. When temperature is rapidly increased, at 44 °C synaptic transmission at the neuromuscular junction ceases and primary sensory neurons stop functioning. Even though animals do not respond to stimuli after being warmed to 44 °C, if sensory neurons are returned to 20 °C saline after two minutes, they may regain function. Conversely, the neuromuscular junction does not regain function after two minutes in 44 °C saline. Examining behavior and heart rate while warming at 1 °C/min, 12 °C/min, or 46 °C/min to 80 °C indicated that at approximately 40 °C the heart rate is altered. Within 10 s at 80 °C, the heart stops with the highest heating rate. Directly placing crayfish in boiling water stopped the heart quickest, within 10 s, which likely represents denaturing of the tissue by heat. Using an impedance measure to detect a heartbeat may also be influenced by movements in the denaturing process of the tissue. A rapid increase in the temperature of the crayfish above 44 °C is key to limit its exposure to noxious stimuli.

## 1. Introduction

General care of animals prior to slaughter and techniques to ensure efficient stunning are continually being investigated to make the process of slaughter for human consumption more humane [1,2,3]. Crustaceans are a substantial source of animal protein for human consumption and crayfish alone represent 12% of all farmed Crustacea, second only to shrimp [4]. China is the biggest producer of crayfish, followed by the United States of America where *Procambarus clarkii* is native, and Europe is third [4]. The growing public interest and scientific progress in the area of animal welfare has brought focus on best practices for stunning crustaceans prior to processing for human consumption [2]. The need to reduce or remove causes of noxious stimuli when caring for crustaceans at all stages of the value chain is driven by new research which brings evidence that this category of animals is considered to be complex in behavior and to have some degree of awareness [5], and some authors even claim they may experience pain [6,7,8]. Although this evidence is not unanimously accepted [2], some policy makers have acted upon and incorporated the findings in new legislation [9]. The argument against the conclusion that crustaceans ‘feel’ pain is rooted in the fact that behavior as a measure for pain perception is not entirely reliable and reproducible [2]. It is only recently that physiological measures in crustaceans have been used alongside behavioral indicators to help draw conclusions on their sensory perception and thus welfare [3,10,11]. The approaches to stun crustaceans with the quickest and the least stressful methods have been examined since the 1950s [12]. Approaches include rapid cooling, rapid and slow warming, exposure to CO_2_, and electrical stunning [2,3,10,13,14]. However, the variety of crustacean species and their adaptation to different environments, as well as their various sizes and shapes, results in a wide array of physiological responses to the stunning methods available, making it difficult to adopt a universal method. A marine shrimp adapted to warm environments (27–28 °C) responds differently than a lobster (*Homarus americanus*) or a blue crab (*Callinectes sapidus*) conditioned to cold (10 °C) when stunned by cold-shock (~4 °C) [3]. The electrical stunning technique requires consideration of varying current strengths for fresh and sea water crustaceans as well as size differences and types of crustaceans [3,13,14].

Studies with large crustaceans suggested that slow heating did not elicit behavioral responses [10]. This has had diametrically opposed interpretations. On one hand, it was considered as an index of discomfort or stress [10]; on the other hand, it has been considered a sign that the animal is not feeling ‘pain’ [12]. However, behavioral responses alone are not effective in determining if the animals have sensory perception, thus making it hard to ascertain whether an animal does not move due to a lack of sensorial perception or due to muscle paralysis. More recently, this topic has been examined with behavioral and electrophysiological measures [3,10]. Lobsters (the American lobsters *Homarus americanus* and the European lobsters *Homarus gammarus*) and crayfish (*Astacus astacus* and *Astacus leptodactilus*) showed baseline electrical activity of the ventral nerve cord around 32 °C, which then decreased to zero as the temperature increased to 40 °C. The procedure was performed by slowly increasing the water from 7 °C to 40 °C at a rate of approximately 1 °C/min [10].

The red swamp crayfish (*Procambarus clarkii*) is a species of commercial interest in the food industry and it is produced in China, the United States, and Europe [4]. Different methods of killing are used around the world and mostly consist of heating or cooking live animals. To determine if a certain rate of heating is faster than others to inactivate neural function, the species’ behavioral response and physiological measures were recorded. We exposed crayfish to different rates of heating to determine how these affect the animal’s sensorial perception

In this study, the word ‘paralysis’ is used to indicate absence of movements but not absence of ECG or sensory nerve activity; the word ‘anesthesia’ is used to indicate absence of ECG or sensory nerve activity with or without presence of movement.

## 2. Materials and Methods

Experiments assessed behavior, heart rate, sensory function, and motor unit function while warming live crayfish or isolated tissue from crayfish. The behavioral experiment was performed on one group of six crayfish. Behavior was observed and recorded while warming the water at 1 °C/min to ascertain when they appeared to be dead, determined by a lack of response to physical stimuli applied to the eye stalk and no movement of the body. This method was the same used by Fregin and Bickmeyer [10] in which crayfish (*Astacus astacus* and *Astacus leptodactilus*) and lobsters (*Homarus americanus* and *Homarus gammarus*) were studied. A second group of six crayfish were wired for recording heart rate while warming the water at 1 °C/min to determine if the observational index of death (no responses to physical stimuli) correlated with an absence of a heart beat and the temperature at which a heartbeat could not be detected. A third set of experiments recorded heart rate and behavioral changes. This group was first acclimated overnight to 10 °C to simulate some aspect of commercial crayfish cooking where they are collected and cooled for transport and storage before being cooked. The water of this group was then heated from 10 °C to 60 °C at a rate of ~12 °C per minute. A fourth experiment recorded heart rate while increasing water temperature at a rate of 46 °C/min from 10 °C to 84 °C in crayfish maintained overnight at 10 °C. Commercial processing of crayfish uses similar rapid heating. A fifth experiment conditioned two crayfish to 10 °C overnight and directly placed in boiling water while recording the heart rate. This method is commonly adopted by restaurants and food venues of crayfish boils or cookouts in the USA.

One subject appeared dead at boiling temperature (~98 °C), but some sporadic non-rhythmic deflections occurred in the recording trace for a heartbeat. A trial was set up to examine if the non-rhythmic deflections were artifacts in the impedance recordings from the heart muscle denaturing or effects on the hemolymph being heated. The heart rate of one crayfish was recorded while at room temperature (20 °C) and after killing by CO_2_. After no heart rate and no response to stimuli were detected, the crayfish was placed directly in boiling water while monitoring the recording used for determining heart activity.

Additionally, isolated abdomens from six crayfish were used to record the sensory nerve activity from the muscle receptor organ (MRO) as a measure of the effects on sensory neurons while changing the bathing saline temperature from 20 °C to 44 °C and maintaining at 44 °C for two minutes prior to returning the saline temperature to 20 °C. This was conducted by pipetting the saline out of the recording chamber and replacing it quickly with saline at the new temperature and repeated to maintain the temperature. Six first walking legs, all from different crayfish, were used for measures of synaptic transmission at the neuromuscular junction using the same heating protocol as for the MRO.

### 2.1. Animals

Red swamp crayfish (*Procambarus clarkii*) were obtained from a food distribution center in Atlanta, GA. These were purchased from a local supermarket in Lexington, KY, USA when still alive. Throughout the study, midsized crayfish measuring 30–62 mm in postorbital carapace length (posterior dorsal surface of the orbital cup to the end of the carapace directly posterior to the eye cup) were used. The measures were made with calipers (Swiss Precision, Newton, MA USA, 02458, 0.1 mm). The animals varied in weight from 39–48 g. They were individually housed in standardized plastic aquaria (33 × 28 × 23 cm, water depth 10–15 cm) with temperature maintained between 20 and 21 °C, weekly water exchanges and fed dry fish food (salinity 25–26 ppt; O_2_ at 7.4–7.6 mg/L).

Care was taken not to use more animals than necessary for these studies. According to University of Kentucky Administrative Regulation (AR) 7:5, oversight applies to “all research, teaching, and testing activities involving vertebrate animals conducted at University facilities or under University sponsorship, regardless of the species or source of funding”. This AR follows The United States Department of Agriculture (USDA) Animal Welfare Act, the PHS Policy, and the *Guide* definition of animal [15]. With this in mind, the Institutional Animal Care and Use Committee (IACUC) review is not currently required in the United States for the use of crayfish in research.

### 2.2. Electrocardiocrams (ECG)

Physiological measures consisted of changes in heart rate (ECG), sensory nerve activity of large neurons in the abdomen, and synaptic transmission at the neuromuscular junction. The heart of the crayfish is neurogenic, meaning that the rate of beating is indicative of the neuronal function [16,17]. Behavior was assessed by monitoring the reflex response to tapping of the eye stalk. We also examined the physiological activity in excised preparations of primary sensory neurons and synaptic transmission at neuromuscular junctions while increasing temperatures from 21 °C to 44 °C as additional measures of neural function.

**Figure 1:**
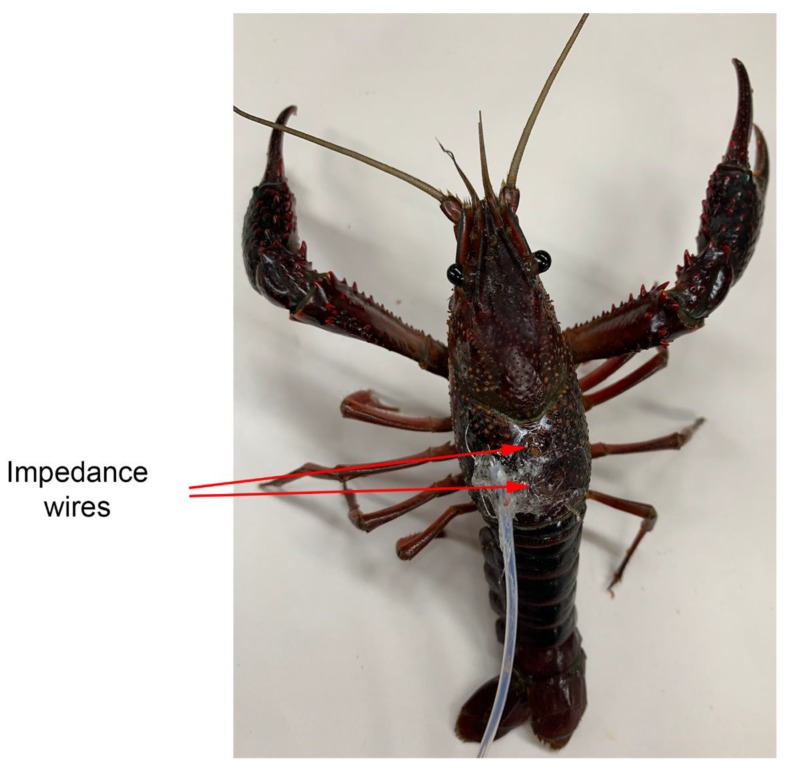
Placement of recording leads for measuring heart rate in an electrocardiogram (ECG) for a crayfish. The ECG leads are placed in an anterior-posterior arrangement for obtaining the best signals.

The procedures and analysis are similar to those described in a previous study investigating cooling of crustaceans [3]. The preparation of the ECG leads is described in detail in text and video format in previous publications [18,19]. In brief, insulated stainless steel wires (0.13 mm diameter; A-M Systems, Carlsburg, WA, USA) were inserted into small holes made in the cuticle (see Figure 1 in [3]). For heart rate measures, the wires were placed through the dorsal carapace directly over the heart [20]. The leads were strung through tubing to prevent the crayfish from damaging the wires and the tubing was glued to the carapace (Figure 1). Once the wire was in place, a small amount of glue (cyanoacrylate ester) and accelerator (HobbyTown USA, Lexington, KY, USA) was placed on the tubing. The use of the fast-drying glue reduces handling stress of the animals [18,19]. To eliminate the risk of damaging internal organs, special attention was made on inserting only a short portion of wire (1 mm).

For ECG recordings, both wires were connected to an impedance detector (UFI, model 2991, 545 Main Street, Suite C-2, Morro Bay, CA 93442, USA) which measures dynamic resistance between the leads. Subsequently, the detector was linked to a PowerLab/4SP interface (AD Instruments, Unit 13, 22 Lexington Drive, Bella Vista, New South Wales 2153, Australia) and calibrated with the PowerLab Chart software version 5.5.6 (AD Instruments). The acquisition rate was set to 1 kHz. The heart rate was calculated by direct counts of each beat over short 10–20 s intervals and converted into beats per minute (BPM). The responsiveness of a sensory-CNS-cardiac ganglion neural circuit was assessed using a glass rod to tap the eye to induce an alteration in the heart rate and for behavioral assessment of the eye stalk withdrawal reflex.

**Figure 2:**
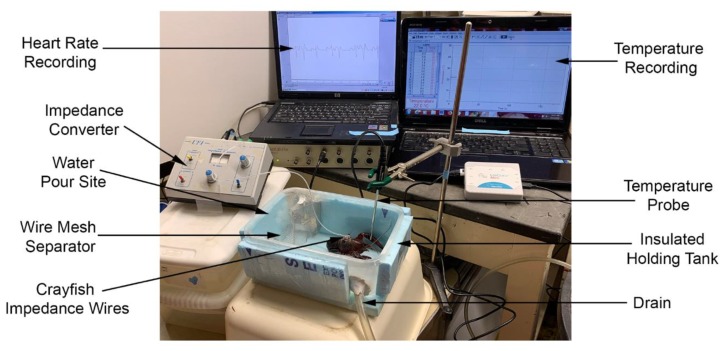
Recroding set up for ECGs while changing the water at various temperatures. The ECG leads were fed into the impedance converter which in turn was fed to an A/D board to record on a computer. The temperature probe was fed to a Vernier A/D converter to then record on a computer. The plastic chamber had insulation around it and a wire mesh on one side placed in the chamber to disseminate the water flow. A drain tube allowed the water to be removed as the new water was added at different temperatures.

The water was changed with an insulated chamber which allowed water to flow in and a drain across from the inlet to allow water out of the chamber. The outlet was regulated with a pinch clamp placed on the outlet hose as water of increasing temperature was added to the bath. Water was added on one side of a fine aluminum screen mesh which dispersed the warmer water into the holding part of the chamber where the crayfish was being monitored. A thermal probe was placed in the chamber close to the location of the crayfish and suspended to the midlevel of the crayfish body. The water was brought to the desired temperatures in a series of beakers prior to experimentation. The temperature was controlled by adding water at higher or lower temperature to the beakers prior to adding to the chamber. A Vernier stainless steel temperature probe was used (resolution: 0.03 °C (0 to 40 °C) and 0.1 °C (40 to 100 °C); Vernier Software & Technology, Beaverton, OR 97005, USA). See Figure 2 for an illustration of the recording set up.

### 2.3. Muscle Receptor Organ (MRO)

**Figure 3:**
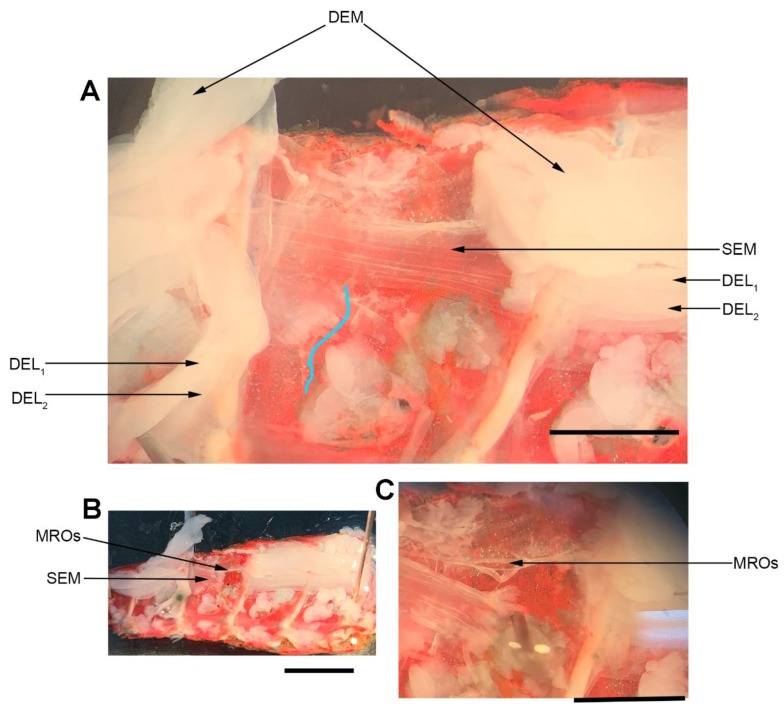
Location of the muscle receptor organ (MRO). (**A**) A hemi-section of the crayfish abdomen viewed from ventral to dorsal after removal of the ventral muscle. The DEM muscle is cut on the right side of figure and pulled to left. The segmental nerve containing the nerves associated with the MRO was taken up by a suction electrode (the blue line outline is the segmental nerve). The deep extensor muscles (DEL1, DEL2, and DEM) and the superficial extensor medial muscle (SEM) are shown. The MRO organ is beneath the DEL1 muscle alongside to the DEM. (**B**) The superficial extensor medial muscle (SEM) was cut on the right side in the figure to expose the two MRO muscles. (**C**) The two MRO muscle fibers are shown with the nerve bundle pulling the fiber toward the SEM muscle. Scale bar: A and C is 2.5 mm; B is 5 mm.

The dissection and recording procedures are described in [21]. In brief, the isolated crayfish abdomen was placed in a Sylgard-lined dish filled with crayfish saline (Figure 3). The abdominal joint was moved using a wooden dowel from a relaxed position to a stretched position in a 1 s (s) time frame, held for 9 s, and then moved back to the starting position. An insect dissecting pin was used to mark the displacement range, and each displacement was marked on the computer recording file. The segmental nerve to the segment of interest was pulled into a suction electrode for recording the extracellular spikes. After the nerve ending was suctioned into the tip, a small amount of clear petroleum jelly was placed around the tip of the electrode to provide a tight fit for the nerve and lumen of the electrode. The tight fit allowed for a better signal to noise ratio.

The movement to the stretched position was made within 1 s. The stretched position was then held for another 9 s. The entire 10 s were used to measure the activity of the neurons. Three trials were performed for each time point. The activity from the set of three trials was averaged for each preparation. Measures were made during saline exposure at 20 °C, two minutes after exchanging the 20 °C saline for 44 °C saline, and after returning the saline to 20 °C. Control experiments were performed with exchanges of saline to saline without altering the temperature for the same times, and the data were presented in previous reports [22]. The crayfish saline used was a modified Van Harreveld’s solution (in mM: 205 NaCl, 5.3 KCl, 13.5 CaCl_2_·2H_2_O, 2.45 MgCl_2_·6H_2_O, and 5 HEPES adjusted to pH 7.4 at 21 °C). The saline was exchanged with warmer or cooler saline, which was maintained in beakers, with some beakers on a hotplate and titrated to others to reach the desired temperature. The saline volume in the recording dish was 15 mL and was exchanged by removing the saline with a 30 mL syringe followed by adding saline of the desired temperature to the bath. The exchange was completed within 30 s.

### 2.4. Neuromuscular Preparation

**Figure 4:**
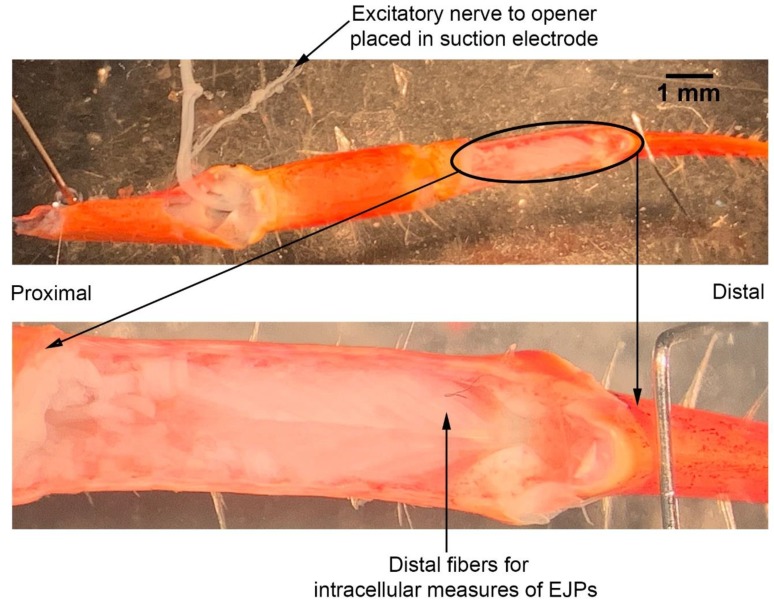
Opener muscle preparation. (**A**) A view of the crayfish walking leg opener muscle and excitatory axon bundle for the muscle. (**B**) The muscle excitatory junction potentials were recorded from the distal muscles of the preparation.

The dissection and recording procedures are described in Cooper and Cooper [23]. In brief, the ventral cuticle of the propodite and the closer muscle is removed to expose the ventral surface of the opener muscle in the propodite cavity (Figure 3). The excitatory motor neuron to the opener muscle, identified in the meropodite segment, is pulled into a suction electrode for stimulation. After the nerve ending was suctioned into the tip, a small amount of clear petroleum jelly was placed around the tip of the electrode to provide a tight fit for the nerve and lumen of the electrode. The tight fit allowed for a lower voltage to be used to stimulate the nerve [24]. To evoke action potentials in the excitatory axon, it was selectively stimulated by a stimulator (S88 Stimulator; Astro-Med, Inc., Pleasanton, CA, USA). The distal muscle bundle of the opener was impaled with sharp intracellular electrode (20–30 mOhm resistance) filled with 3 M KCl (Figure 4). The excitatory junction potentials (EJPs) were recorded from the muscle fiber of interest. Short term facilitation in the EJPs was obtained by stimulating at 40 Hz for 25 stimuli within a train and repeated every 5 s. A standard head stage and amplifier for intracellular recording was used (Axoclamp 2B, and 1 X LU head stage, Molecular Devices, Sunnyvale, CA, USA). The crayfish saline used was the same described above for the MRO preparation. The saline was exchanged with saline of the desired temperature in the same manner as for the MRO preparation as stated above.

### 2.5. Statistical Analysis

All data were expressed as raw values or a mean (± SEM). The rank sum pairwise test or a sign test (non-parametric) was used to compare the differences in the heart rate, MRO activity, and the presence of EJPs with exposure to various temperatures. The non-parametric test was used because the data were not normally distributed, as there was no activity to measure in some conditions. A *p*-value of ≤0.05 was considered statistically significant. SigmaPlot (version 14.0; Systat Software Inc. San Jose, CA, USA) was used for the analysis.

## 3. Results

### 3.1. Effect of Warming on Behavior

The gradual warming of the water at ~1 °C/min starting from 20 °C resulted in the crayfish attempting to climb out of the chamber at around 36–38 °C in all 12 animals tested. At 40 °C the crayfish laid sideways and the walking legs twitched for 30–60 s (*N* = 12, *p* < 0.01 nonparametric sign test, two-tailed). This first set of six crayfish, which were not wired for monitoring ECG, appeared physically dead at 40 °C as the eyes were white and no response occurred from tapping the eye stalks, pinching the tail, or picking the animal up. After a minute at 40 °C, the crayfish appeared to have died. They were removed and placed in holding containers with water temperature at 20–21 °C. The crayfish had no obvious responses to eye stalk touching or handling (*N* = 6, *p* = 0.03 nonparametric sign test, two-tailed). The limbs were limp and dangling. None of these animals appeared to show any movements and did not recover in the following hour. These were determined to have died. The eyes and body muscle as seen on the ventral side of the abdomen were white.

### 3.2. Effect of Warming on Cardiac Function

**Figure 5:**
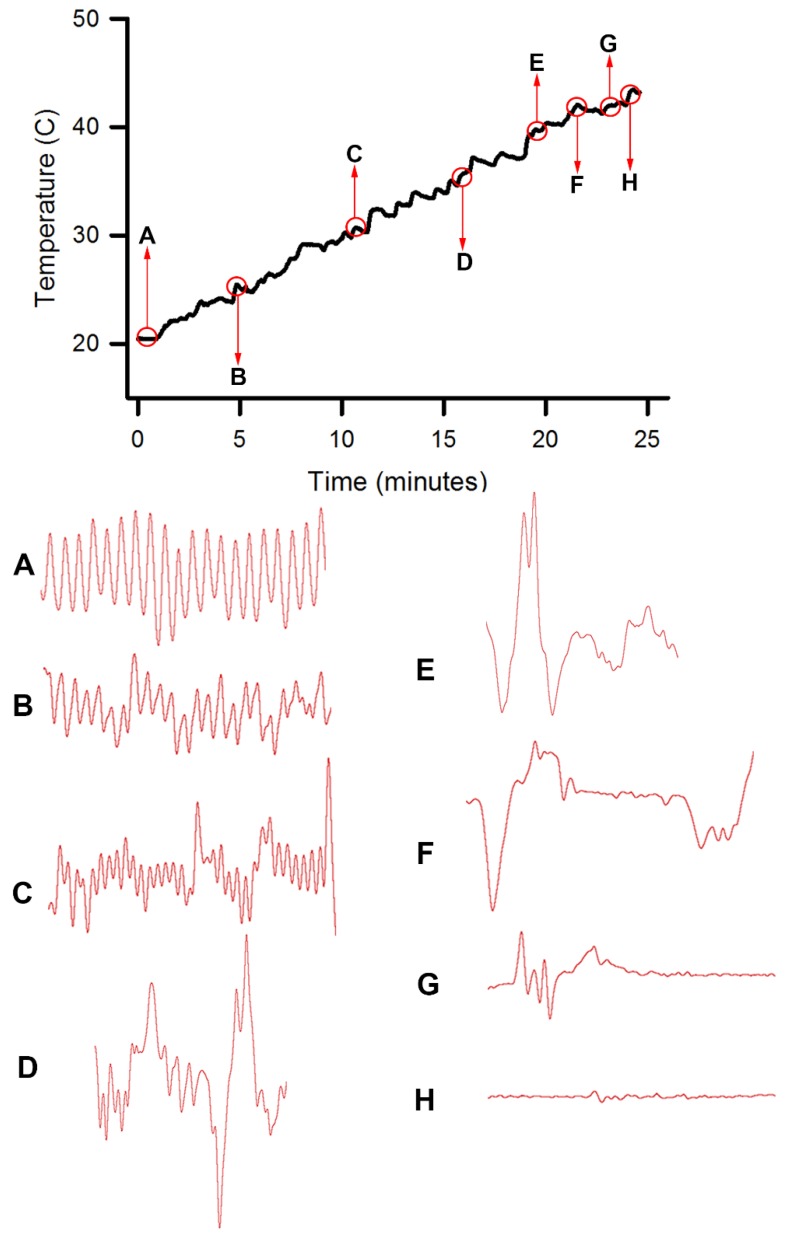
Heart rate measures at various water temperatures starting at 21 °C and increasing at ~1 °C per minute. The ECG traces are shown at the various time points while the temperature was increasing. The letters in the top trace correspond to the traces shown below in alphabetical order. Each trace is 10 s in duration.

The second set of six crayfish were wired and their ECG was monitored as the water temperature was raised. This was performed in the same manner as for the first set of crayfish. Since it was noted that a heartbeat was still present at 40 °C, although slowed and sometimes arrhythmic, the rise in the water temperature was continued to 44 °C. At 44 °C the heartbeat slowed, became sporadic, and then stopped (Figure 5; *N* = 6, *p* = 0.03 nonparametric sign test, two-tailed). The water was then cooled, but the heartbeat did not return and the animals did not recover over the next 30 min. The eyes and body muscle as viewed the on ventral side of abdomen were white. These crayfish were determined to have died.

For both sets of animals, a light touch on the eye was performed as the temperature of the water was raised. The eye withdrawal stopped at 40 °C in all 12 animals (*N* = 12, *p* < 0.01 nonparametric sign test, two-tailed). This suggests that exposure to 40 °C water results in paralysis (lack of response to stimuli), followed by death at 44 °C. Between 40 °C and 44 °C crayfish in the first experiment did not respond to touch, but heartbeat alterations were detected in response to touch, suggesting a state of paralysis.

**Figure 6:**
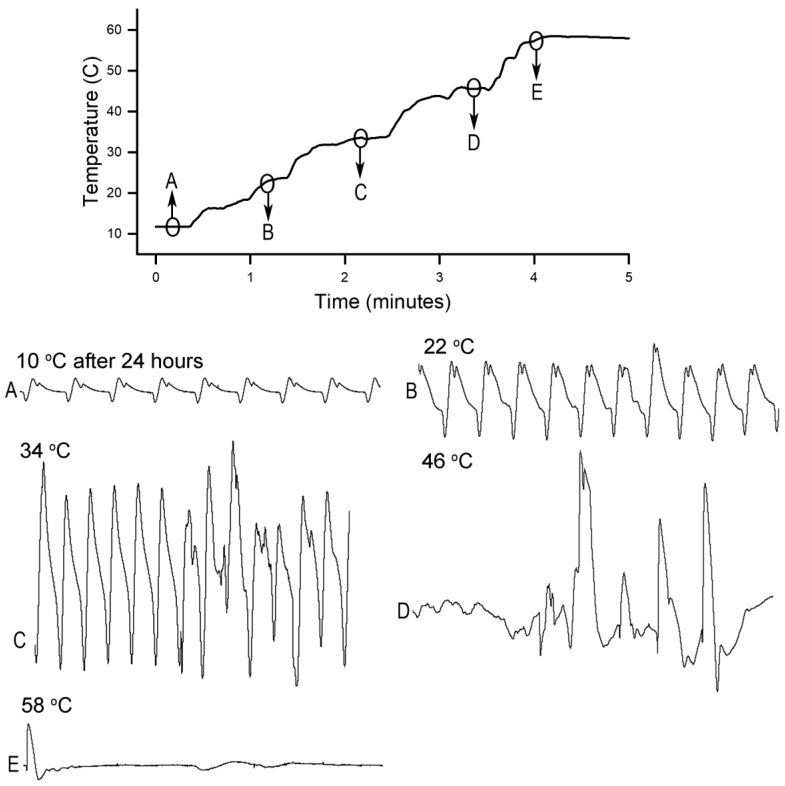
Heart rate measures at various water temperatures starting at 10 °C and increasing at ~12 °C per minute after conditioning to 10 °C for 24 h. The ECG traces are shown for crayfish after 24 h of being maintained at 10 °C prior to the heating and at each subsequent 12 °C interval during the heating to 60 °C. The flat line in the recording occurred at 58 °C. The sequence of events is in alphabetical order. Each trace is 10 s in duration.

To better simulate some forms of commercial cooking, crayfish were acclimated to 10 °C overnight for 24 h prior to being heated. Thus, we chose two more rates of warming to examine, one at 12 °C/min and one at 46 °C/min. Three crayfish underwent the 10 °C conditioning and were then placed in water heated at ~12 °C per minute from 10 °C to 60 °C while recording the ECG (Figure 6). The impedance recording produced a flat line within a minute at 60 °C in all three crayfish. All temperature measures here presented relate to that of the water the crayfish were in.

**Figure 7:**
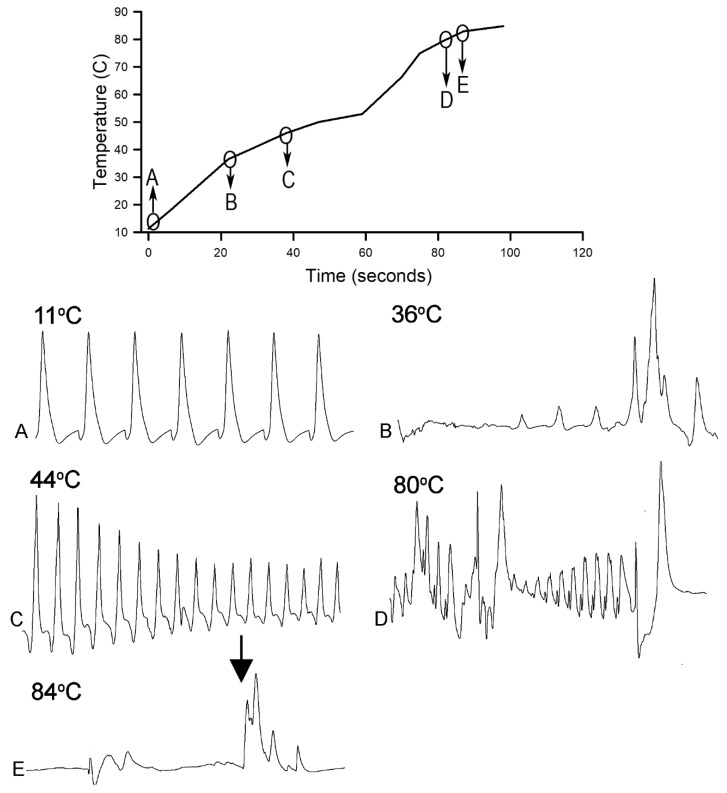
Heart rate measures at various temperatures while increasing from 10 °C to 84 °C in crayfish conditioned at 10 °C for 24 h. The ECG traces are shown for crayfish after 24 h of being maintained at 10 °C prior to the heating and during the heating at a rate of 46 °C/min. The heart rhythm stopped at 80 °C. The arrow shown in E represents artifacts from re-zeroing the impedance detector while recording. The sequence of events is in alphabetical order. Each trace is 10 s in duration.

In commercial settings, crayfish are caught from the rice fields or lakes they live in and are then either processed directly, or can be kept in a cold storage overnight where their body temperature drops to about 10 °C. Crayfish are cooked by exposing them to hot temperatures (>70 °C) to ensure product safety. Cooking can be done by immersion into hot or boiling water, or through steaming. In either case, it takes some time before the crayfish body temperature reaches that of the cooking medium. We simulated such heating with three crayfish that underwent the 10 °C conditioning for 24 h and then heated in water at a rate of 46 °C/min from 10 °C to 84 °C while recording the ECG (Figure 7). The two crayfish died at 80 °C. A complete flat line in the recording occurred after 68 s for one crayfish and after 38 s for the second crayfish after the water reached 80 °C.

**Figure 8:**
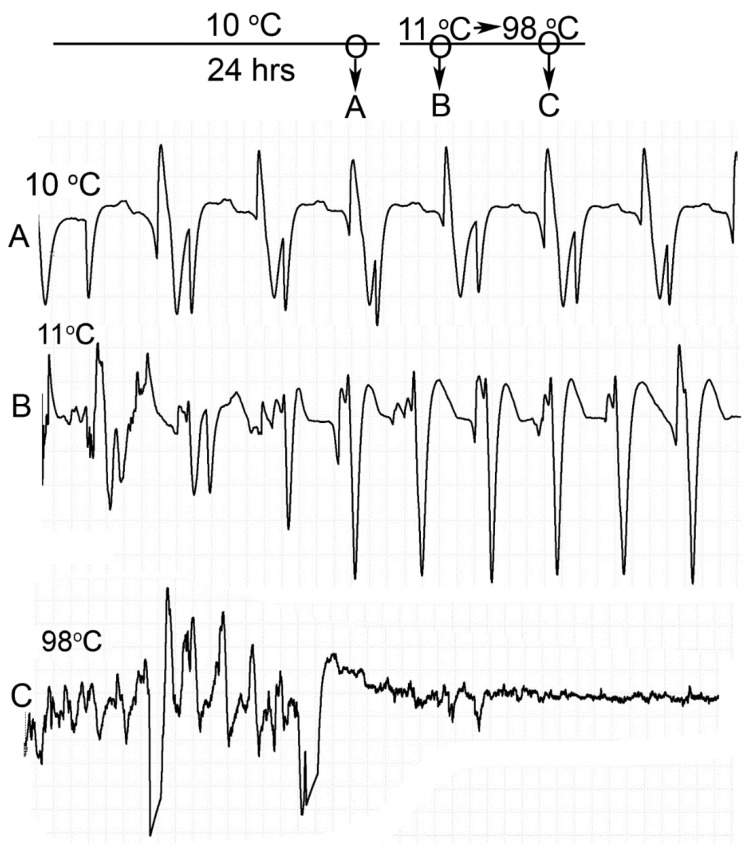
Heart rate measures at 11 °C and boiling water at 98 °C. The crayfish was conditioned to 10 °C for 24 h. (**A**) A representative ECG trace of a crayfish placed in a container at 10 °C after 24 h. (**B**) The crayfish was moved from one container to another with water at 11 °C to determine if the ECG trace was maintained with movement. (**C**) The impedance began recording immediately when placing the crayfish into the boiling water. Each trace is 20 s in duration.

Two crayfish were placed directly in boiling water after 24 h of being maintained at 10 °C. To determine if picking the crayfish up and placing it into a new container produced an alteration in the impedance measure, obscuring the heart rate or causing artifacts in the recordings, the crayfish were moved from one container at 11 °C to the another container also at 11 °C and then placed directly into 98 °C boiling water. The heartbeat was readily detected and was maintained when moving the crayfish from one container to another at 11 °C (Figure 8). Upon placing the crayfish directly into boiling water, the rhythmic heart rate was observed for five seconds followed by non-rhythmic deflections in the impedance trace (Figure 8).

### 3.3. Artifacts within the Impedance Measures with Muscle Denaturing

**Figure 9:**
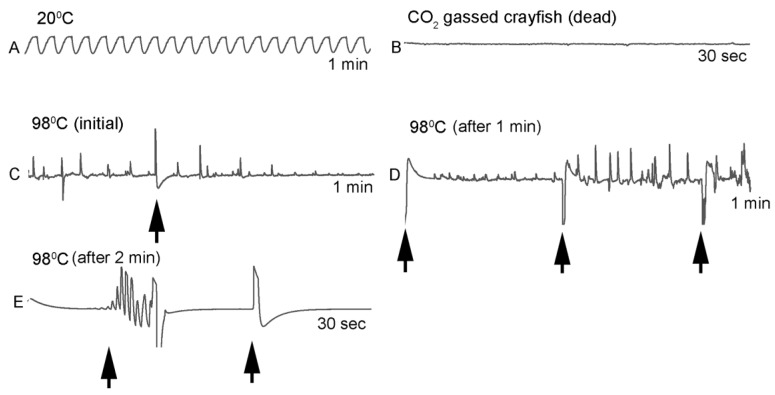
Impedance measures of a single crayfish. (**A**) Impedance measure of the heart rate in the live crayfish for 1 min at 20 °C. (**B**) A 30 s impedance measure of the heart rate of the crayfish after being euthanized with CO_2_. (**C**) Impedance measure of the dead crayfish placed in boiling water in the first min for 1 min and (**D**) after 1 min. (**E**) After 2 min in boiling water the impedance traces flatlined. The arrows shown represent artifacts from re-zeroing the impedance detector while recording. The sequence of events is in alphabetical order.

In the experiments rapidly reaching a temperature of 80 °C and higher, the crayfish appeared dead based on visual inspection with opaque eyes, no response to touch stimuli, and the abdomen residing in a stiff flexed position, but some deflections in the impedance traces occurred for brief moments. The abdomen in a flexed position is of interest as this suggests the large muscle contracted and became denatured with heat in this position. The heart may also undergo contraction and movement during the denaturing. If so, the deflections with the impedance measures, which do not show a rhythmicity, might be due to the denaturing process of the cardiac muscle. To address this potential issue, the heart of one crayfish was recorded with the impedance technique and then subsequently gassed with CO_2_ until the heartbeat stopped and the animal was limp with legs and chela dangling when picked up. In this state, the animal was placed in boiling water while recording the impedance during the denaturing process of the body muscles as well as the heart. The impedance traces after CO_2_ gassing did not illustrate any deflections, but the rapid heating in boiling water did produce transient deflections in the recording which returned to a flat line after 2 min. Figure 9 illustrates the results of this experiment.

Taking into consideration that boiling a dead crayfish resulted in arrhythmic deflections in the traces for impedance measures, the timing of death was determined when a rhythmic pattern in the heartbeat was no longer measured for the different heating conditions (Table 1).

### 3.4. Effect of Warming on Primary Sensory Neurons

**Figure 10:**
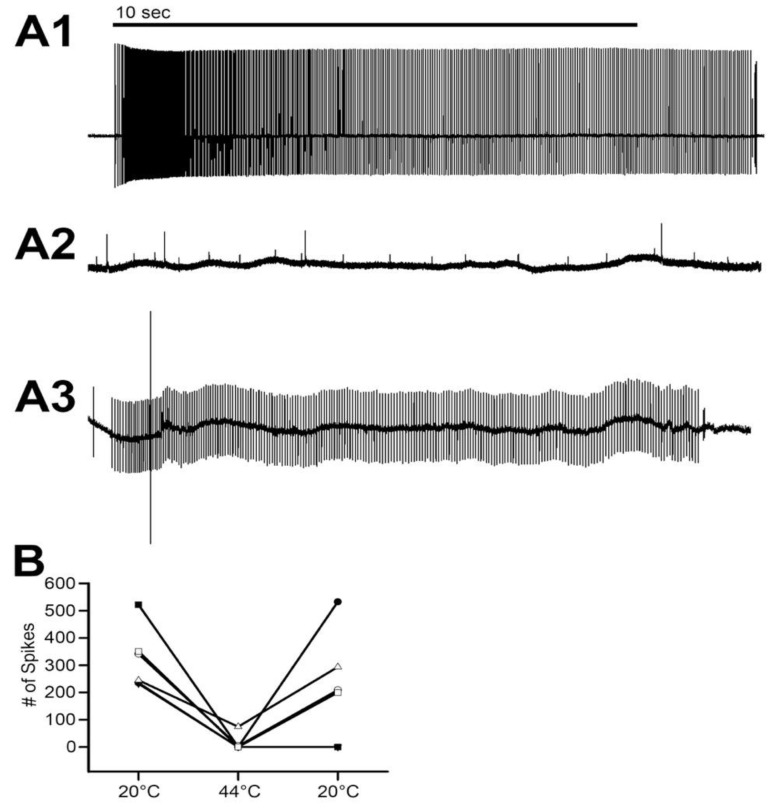
The activity of the MRO at various temperatures. (**A1**) Representative trace of the extracellular 10 s activity of the MRO segmental nerve in saline at 20 °C. (**A2**) Representative trace of the extracellular 10 s activity of the MRO segmental nerve at 44 °C. (**A3**) Representative trace of the extracellular 10 s activity at 20 °C after being at 44 °C for 1 min. (**B**) The mean number of spikes for the 10 s activity for each MRO preparation at each temperature. The heating to 44 °C had a significant effect in reducing the neural activity (N = 6, *p* < 0.05 non-parametric sign test). Each line represents individual preparations and some of the symbols overlap.

The primary sensory activity of the MROs ceased when the saline temperature was increased from 20 °C to 44 °C in all six preparations (Figure 10; *N* = 6, *p* = 0.03 nonparametric sign test, two-tailed). After being exposed to 44 °C for two minutes and returned to 20 °C, four preparations showed a return of activity when stimulated by moving the abdominal segment (Figure 10).

### 3.5. Effect of Warming on Synaptic Transmission at Neuromuscular Junction

**Figure 11:**
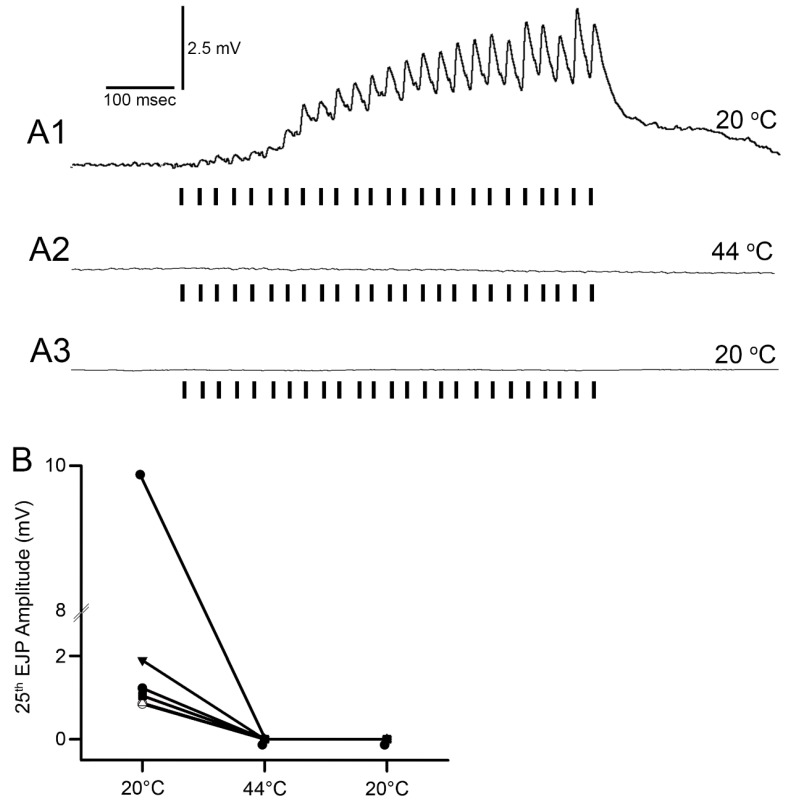
The excitatory junction potentials (EJPs) recorded from the opener muscle at various temperatures. (**A1**) Representative trace of the EJPs recorded with an intracellular electrode from the distal muscle fibers in opener muscle of a crayfish walking leg at 20 °C. The responses show a marked facilitation that occurs throughout the stimulation train delivered at 40 Hz for 25 stimuli. (**A2**) The EJPs are not able to be observed at 44 °C. (**A3**) Upon returning the preparation to 20 °C, the EJPs are still absent. The vertical bars represent the 25 stimuli pulses. (**B**) The mean 25th EJP amplitude (mV) for each opener muscle preparation at each temperature is shown (N = 6, significant difference between 20 °C and 44 °C, *p* < 0.05 paired *t*-test).

The EJPs of the opener muscle ceased at 44 °C for all preparations (Figure 11; *N* = 6, *p* = 0.03 nonparametric sign test, two-tailed). After being exposed to 44 °C for two minutes and returned to 20 °C, the responses did not return. Five of the preparations stopped producing EJPs abruptly by 35 °C, with only one preparation showing a gradual reduction in the EJP amplitude as temperature was increased.

## 4. Discussion

This study highlighted the behavioral and physiological effects of warming live crayfish (*P. clarkii*) to various temperatures at multiple rates. Behavioral assays of being limp or no longer responding to stimuli, such as the eye stalk tapping, do not indicate vital organ function of the heart. Recording heart rate with an impedance measure reduces baseline noise, but artifacts from potential cardiac muscle movement during denaturing caused by heat produces displacements in the impedance recording. By heating a dead crayfish while recording impedance measures in the same manner, the type of displacements in the electrical traces are able to be distinguished and compared to the recordings made with live crayfish. Monitoring the heart rate at slow heating rates (~1 °C/min) resulted in the heartbeat losing rhythmicity around 40 °C. At a ~12 °C/min rate of temperature increase, the heartbeat lost rhythm at 51 °C on average, and when a rate of ~46 °C/min was used the crayfish lost heartbeat rhythm within 6–9 s after reaching 80 °C. In placing crayfish conditioned at 10 °C directly in boiling water (98 °C), the heart rate rhythm was lost within 12 s. As for the excised tissue of sensory and neuromuscular junctions, a short bout of high temperature to 44 °C does not necessarily cause sensory neurons to die immediately if the temperature is quickly returned to cooler temperatures. However, the neuromuscular junctions after being heated to 44 °C for 2 min did not regain synaptic function when quickly lowered to 20 °C. This may suggest that some of the animals were paralyzed for a short time before dying, as all of the whole animals heated to 44 °C died.

All of the initial set of intact animals, for which heart rates were not recorded, died after they were returned to 20 °C after being heated to 40 °C. Thus, although dead, it is not known how long the hearts may have continued to beat after being returned to 20 °C water.

Since the crayfish heart is neurogenic, the heart still beating at 40 °C indicates the central and cardiac ganglion neurons were still functioning despite a lack of response to stimuli. Future studies could investigate how long the heart would maintain a neurally-controlled beating rhythm. Since the animals did not physically recover from the 40 °C exposure, we did not see the need to investigate this matter any further. The neural control to the heart ceased at 44 °C within a two-minute period and did not recover when returned to a cooler environment. Thus, for this species of crustacean, water temperatures at or above 44 °C should be sufficient to stop the neural control of the heart. Faster heating rates, 12 °C or 46 °C/min, would allow the crayfish to maintain a rhythmic heart rate at a higher temperature, but beating will stop in a shorter period of time. The boiling of crayfish, as might be expected, was the fastest means of stopping any detectable rhythm in the heart. These different temperatures at which the heartbeat stops could reflect the fact that different rates of water heating have a different rate of warming up of the body of the animal. The faster the water temperature increase, the faster the rise in body temperature of the animal and therefore the faster the critical temperature of muscle and heart denaturing will be reached.

In addressing neural function, the absence of activity from primary sensory neurons at 44 °C is informative. It is reasonable to assume these sensory neurons are representative of other sensory neurons and central neurons in regard to heat inactivation of the neuron. The short 2-min exposure at 44 °C only resulted in two of the six preparations being unable to regain function when quickly cooled back to 20 °C. It was not determined how long these sensory neurons would have been able to maintain function after being cooled back to 20 °C. However, since the muscles and the segmental nerves in the abdomen turn an opaque white from the heat exposure, it is expected that the cells would die soon afterwards. The opaque white color is due to damage (coagulation of proteins) occurring when crustacean muscles are heated. The difference in the muscle consistency from a healthy cell being damaged physically by a cut and one being slowly heated is that the heated muscle fibers can be transected and the cut ends remain in place. A slightly injured muscle cell will turn white and sometimes twitch and then remain in a contracted state. The heated preparations made excellent models for dissection; the muscles were maintained in position and could be pinned to one side to show attachment points or other structures, such as the MROs within the abdomen.

The mechanism to explain why the neuromuscular junctions of the opener muscle cease to function appears to be due to a reduction in the ability of the presynaptic nerve to generate the synaptic release, as indicative of the reduced amplitude of the EJPs. The silencing of the response was permanent after 2 min at 44 °C. However, if the postsynaptic glutamate receptors or ion channels of the muscle are being compromised during the warming, the amplitude could be reduced due to postsynaptic alterations. Given that a reduced EJP amplitude was recorded, it is likely that the motor nerve is able to be evoked and that it was not a failure in electrical activation or conduction of the motor nerve. However, at 44 °C, all preparations ceased in being able to respond to stimulation of the motor nerve. In addition, the opener muscle, just as the muscle in the abdomen, turned an opaque white in color within 2 minutes of exposure. The resting membrane potentials were also not able to be maintained and depolarized rapidly. The original membrane potentials did not return upon cooling but instead remained depolarized, indicating the cells died from being denatured by the heat exposure.

Slow warming from 21 °C to warmer temperatures may inactivate neurons by making it harder to reach a threshold to initiate an electrical signal. When considering that the equilibrium potential for potassium is dependent on temperature, an increase in temperature would theoretically produce a more negative resting membrane potential and neurons may decrease in activity. However, the threshold of activation of a neuron can be lowered if a cell has some sodium channel inactivation at rest that is then removed by making the cell more negative in potential. Since the resting membrane potential is driven by the equilibrium potential for potassium ions, the membrane potential parallels the effects with physiological changes in temperature. Studies in differing species of crayfish (*Astacus leptodactylus*) and crab (*Ocypode ceratophthalma*, *Carcinus maenas*, *Cancer pagurus*) all show a reduction (i.e., more hyperpolarized) in membrane potential with acutely increasing temperature. The relative change is approximately 1 mV to 1.3 mV/1 °C change [25,26,27,28]. However, in an earlier study, hyperpolarization of the muscle membrane potential in *P. clarkii* occurred when cooling the tissue from 20 °C to 10 °C [29,30]. The reason for this change in membrane potential has not been explicitly addressed in prior reports, but it is likely related to the more negative equilibrium potential of potassium (E_K_), since the membrane is more permeable to K^+^ and the resting potential is driven mostly by the E_K_. When the cell membrane and/or channels are compromised, possibly by heat-induced denaturing, physiological processes are not being maintained and many are being altered at once which affects function from individual cells to the whole animal. It is established that membrane resistance is altered with changes in temperature and this also has an impact on excitability of the cells [29,31,32,33,34]. Thus, ion channel denaturing can inhibit neurons and muscles from initiating and conducting electrical activity.

These experiments demonstrate that using heat to kill the red swamp crayfish exposes the animal to discomfort for a period of time which varies from a minimum of a few seconds (8.5 s on average in this experiment) to several minutes depending on the heating method. Whether these methods are considered humane, or if one is more humane than the other, depends on the law and personal ethical choices. The opinion on whether crustaceans are sentient beings and thus capable of suffering and feeling pain is not unanimous. Håstein et al. [35] points out that “sentience, is usually considered a precondition for animal welfare concerns” and a lack of convincing evidence is the reason why several countries have not yet included this category of animals under animal welfare regulations. However, proof of sentience is not the only reason why crayfish should be spared any avoidable discomfort. Animal welfare deals with ethics. From an ethical viewpoint, one of the arguments put forward is that once animals are under the care of humans, then humans have responsibilities towards them which encompass their welfare [35]. For instance, the New Zealand Animal Welfare Act 1999 recognizes crayfish as being sentient animals and, through the New Zealand Code of Welfare for commercial slaughter [9], prohibits the live boiling of crustaceans without prior stunning. In Norway, the Animal Welfare Act applies to decapod crustaceans as well. However, protection is afforded on the basis that animals have intrinsic value and shall therefore be treated well and protected from danger and unnecessary stress and strain. In locations such as Norway, New Zealand, Switzerland, and New South Wales, the heating methods here used would not be allowed for the killing of crayfish. In the absence of laws that protect crayfish welfare, if an operator or consumer takes the stand that crayfish have intrinsic value and should be afforded protection, then the results of this paper show that cooking crayfish in boiling water is more ethical because it minimizes exposure to noxious stimuli.

Stunning or slaughter processes are considered humane when they result in loss of consciousness and sensibility without pain, or instantaneous death [5]. Crayfish are poikilothermic and their heart is neurogenic; thus, they can be rendered insensible through chilling. Loss of consciousness can also be achieved through electric stunning or the use of anesthetics. In fact, stunning through chilling to below 4 °C prior to slaughter is recommended by the New Zealand Code of Welfare for commercial slaughter [9]. A study by Weineck et al. [3] demonstrates that exposing the red swamp crayfish to freshwater below 4 °C for 2 min dampens the perception of external stimuli, but 5 min of exposure stopped the heartbeat and resulted in death for some. In commercial facilities, it was found that if crayfish are cooked after death rather than cooking them live, they differ in appearance. Further studies could look at the possibility of stunning crayfish through cold shock followed by cooking. It may be that if the crayfish are cold enough, a rapid exposure to the heat of the cooking process prevents them from regaining consciousness before death occurs. An assessment of using cold shock to stun or kill crayfish before cooking must also consider the effects on product quality, specifically on appearance.

Two studies [3,10] showed that crayfish could be rendered unconscious through electric stunning. In both studies, electroshock had to be applied for 5–10 s for it to be effective and minutes after electroshock was given, crayfish recovered. While studies confirm that in laboratory conditions electric shock of crayfish is technically feasible, further research and development is needed to adapt electric stunners to the reality of commercial plants and to give meaningful results based on large numbers of animals [13,14] Furthermore, it is not yet known what the effect on product quality may be. The installation and operation of an electric stunner requires considerable investment as well as trained personnel. Funding would help progress the knowledge on this topic and elucidate the commercial applicability and benefits, if any, of such technology to crayfish welfare compared to the traditional and more widely used process of boiling.

Crayfish can also be rendered insensible by exposure to anesthetic agents such as isoeugenol and tricaine methanesulfonate (MS-222) [35] and then killed with heating. Some non-governmental organizations (NGOs) suggest that the use of anesthetic agents before slaughter may be a viable option for avoiding alleged pain and distress. The anesthetization of crayfish with these chemicals may be useful for the improvement of crayfish welfare when these are to be euthanized for laboratory experiments; however, the use of these agents on animals for food consumption has a fragmented landscape of approval. Some of these chemicals are either forbidden to be used on animals destined for human consumption or require considerable withdrawal time which would likely compromise product integrity and safety. For example, in the USA, using isoeugenol is not allowed due to carcinogenic concerns and MS-222 requires a withdrawal period of 21 days. In Norway and New Zealand, isoeugenol is approved for use on animals destined for human consumption and minimal or no withdrawal time is required. The use of chemicals to improve welfare of animals before slaughter could have impacts on product quality and consumers’ perceptions and both aspects should be further investigated.

## 5. Conclusions

In the context of sentient animals, stunning and slaughter are considered humane when they result in loss of consciousness or instantaneous (meaning in less than a second) death [5]. Although the scientific community is not yet in agreement of whether crustaceans are sentient beings or not, it is accepted that the ethical choice for the industry is to strive to handle these animals in the way that causes the least distress. When it comes to killing crustaceans, including crayfish, the general consensus from authorities and NGOs is that live boiling is likely to cause suffering and should be avoided. This paper shows that slowly heating *Procambarus clarkii* causes the animal to try to escape from its environment at about 36–38 °C in all 12 animals tested. This is a clear sign of perception of noxious stimuli [36]. After heating, the neuromuscular junctions decrease in function, likely resulting in paralysis of the animal for a few minutes while sensory neurons are ceasing to function. If the water temperature is increased to at least 44 °C for two minutes, the NMJs do not regain function upon rapid cooling, although some sensory function may return upon cooling. Our data do not support slowly warming crayfish as a humane killing method [5], as they appear to be stressed for a period of time. However, viable alternatives for stunning crayfish before cooking in commercial settings are limited. In jurisdictions where boiling live crayfish is legal, when functional and affordable stunning methods are absent, crayfish welfare at the time of cooking can be improved by using cooking methods that quickly reach and maintain body temperatures above 44 °C. It is shown here that exposing crayfish to a temperature of 98 °C achieves death within an average of 8.5 s. While this is not an instantaneous death, and as such does not meet the definition of ‘humane killing’ [5], it does result in the shortest exposure time to noxious stimuli.

## Figures and Tables

**Table 1:** Temperature and time at which cardiac rhythm ceased with heating.

Test	Description	Crayfish Temperature (°C) before Heating	N = Sample Size	Time to Cessation of Cardiac Rhythm (Seconds). Average +/− SD
1	Temperature increase rate 1 °C/min	20	5	1391 +/− 61
2	Temperature increase rate 12 °C/min	10	3	205 +/− 26
3	Temperature increase rate 46 °C/min	10	2	99 +/− 2
4	Abrupt increase by insertion into boiling water at 98 °C	10	2	8.5 +/− 5
